# Humor in Psychiatry: Lessons From Neuroscience, Psychopathology, and Treatment Research

**DOI:** 10.3389/fpsyt.2021.681903

**Published:** 2021-05-28

**Authors:** Philipp Berger, Florian Bitsch, Irina Falkenberg

**Affiliations:** ^1^Department of Neuropsychology, Max Planck Institute for Human Cognitive and Brain Sciences, Leipzig, Germany; ^2^Department of Psychiatry and Psychotherapy, Philipps-University Marburg, Marburg, Germany; ^3^Center for Mind, Brain and Behavior, Philipps-University Marburg, Marburg, Germany

**Keywords:** humor, psychopathology, well-being, humor intervention, psychiatric disorders

## Abstract

Humor is a ubiquitous human characteristic that is socially motivated at its core and has a broad range of significant positive effects on emotional well-being and interpersonal relationships. Simultaneously, however, impairments in humor abilities have often been described in close association with the occurrence and course of neuropsychiatric disorders, such as schizophrenia, social anxiety, or depression. In the past decade, research in the neuroimaging and psychiatric domain has substantially progressed to (i) characterize impaired humor as an element of psychopathology, and (ii) shed light on the neurobiological mechanisms underlying the role of humor in neuropsychiatric diseases. However, (iii) targeted interventions using concepts of positive psychology have revealed first evidence that a systematic training and/or a potential reactivation of humor-related skills can improve rehabilitative outcome in neuropsychiatric patient groups. Here, we sought to integrate evidence from neuroscience, as well as from psychopathology and treatment research to shed more light on the role of humor in psychiatry. Based on these considerations, we provide directions for future research and application in mental health services, focusing on the question of how our scientific understanding of humor can provide the basis for psychological interventions that foster positive attitudes and well-being.

## Introduction

*Humor* is a unique aspect of everyday human interaction and communication with substantial implications for a variety of variables underlying positive social and emotional functioning (1). For instance, humor might be crucial for the quality of relationships with other people (1), social support (2), attractiveness (3, 4), psychological well-being (5, 6), and coping abilities (7, 8). Besides these well-established positive influences, *impairments* in humor have been described as a core feature of social functioning deficits identified across disorders in the affective and psychotic spectrum (9). Together, these considerations underline a potentially important role of humor for mental health, as first noted in pioneering descriptions of psychiatric conditions (10). It was only in the past decade, however, that a first wave of clinical long-term interventions relying on concepts of positive psychology have begun to systematically use the positive aspects of humor to target social and emotional functioning in patients with mental disorders (11–13). While first evidence speaks in favor of the efficacy of such interventions in the psychiatric domain (13), the further development of systematic interventions targeting humor abilities might be a valuable enterprise. In a narrative review, we sought to integrate evidence from neuroscience, as well as from psychopathology and treatment research to shed more light on the role of humor in psychiatry. Based on these considerations, we provide directions and perspectives for future research and application in mental health services, focusing on the question of how our scientific understanding of humor can provide the basis for the further development of psychological interventions that foster positive attitudes and well-being.

## The Influence of Psychopathology on Humor

For patients with neuropsychiatric disorders, such as schizophrenia or major depressive disorder (MDD), the theoretical notion of deficits in humor abilities has a long tradition (10). In the last two decades, empirical research has progressed to outline alterations in cognitive and emotional components of humor among patients with psychiatric disorders. To examine the complex phenomenon of humor in clinical populations, these studies have used a combination of established measures, including self-report assessments to indicate the patient's “sense of humor” (5, 14) and the use of humor in everyday social interactions (15, 16), and experimental measures to investigate how patients perceive, enjoy, and produce humorous content (17–19). In sum, self-report and experimental studies typically show a tight link between conditions of negative mood states, as prominently observed in MDD or social anxiety, and the aberrant perception of humor could be revealed in a broad range of observations (6, 16, 17, 20), suggesting negatively biased processing of positive stimuli in patients with mood disorders (21). Supporting this notion, the *use* of adaptive (16) humor in everyday social interaction has been observed to be inversely related with vulnerability to depression (15, 16). In patients with schizophrenia, a reduced capacity to experience pleasure is viewed as a cardinal symptom (22, 23) and research addressing patients' humor abilities revealed substantial impairments in cognitive and emotional components of humor (5, 19, 24). These impairments, however, were primarily related to depressive symptoms frequently observed in chronic patients with schizophrenia (25). For example, in a study by Falkenberg et al. (5), the ability to adequately respond to humor (14) and the use of humor as a coping strategy (26) were related to depression scores, as opposed to the positive or negative symptoms *per se*, as observed in patients with schizophrenia. In sum, these results suggest that impairments in humor processing might be specifically related to negative mood states observed in a broad range of psychiatric disorders ranging from depression to schizophrenia. Thus, a big challenge for future research in the neuropsychiatric domain is to identify which neural mechanisms underlie the transdiagnostic entity of humor impairments and how to address these with targeted clinical interventions.

## The Neural Basis of Humor

To enhance our understanding of how individual variation influences the use and processing of humor (both in health and in illnesses), the scientific community studying humor has largely shifted toward the investigation of neurobiological substrates. Using functional Magnetic Resonance Imaging (fMRI) techniques, these studies provide novel and important insights into the brain regions and networks underlying humor abilities as well as the modulating influence of psychopathology. In healthy subjects, the neural mechanisms underlying the processing of humor may converge toward the involvement of two distinct functional networks, that are assumed to be crucial for both the cognitive and emotional aspects of humor. The cognitive aspect of humor is thought to encompass language-based comprehension and recognition processes, eliciting activation in frontotemporal brain regions related to stimulus-dependent language processing (27). Conversely, however, the emotional responses related to phenomena such as amusement and mirth characteristically involved in the appreciation phase of humor, have been found to be associated with increased activation in mesocorticolimbic brain areas, such as the amygdala and insula, as well as the ventral and dorsal striatum (27, 28). Interestingly, the same brain regions have been shown to be essential for the processing of pleasure and reward (29–31) and dysfunction of these regions has been associated with anhedonia in different psychiatric disorders (32), highlighting the role of humor as a vital aspect of enduring positive emotional responses.

Although impairments in humor abilities have been shown to be related with a broad range of negative implications for social and emotional functioning (7, 33), there is still a fundamental lack of knowledge regarding the underlying neural mechanisms of impairments in cognitive and emotional aspects of humor in patients with neuropsychiatric disorders. In a first investigation, Adamczyk et al. (34) have investigated the neural basis of verbal humor comprehension in a sample of patients with schizophrenia with the help of fMRI techniques. The results of this study suggest that patients with schizophrenia exhibit frontotemporal hypoactivation during the cognitive processes related to humor comprehension (34). In another recent investigation, we investigated whether patients with schizophrenia also show hypoactivation in brain regions associated with the emotional component of humor. Using fMRI in combination with a humor processing paradigm, we found alterations in frontostriatal connectivity in patients with schizophrenia, pointing toward the involvement of aberrant frontostriatal coupling as an important mechanism underlying the identified impairments in humor perception defined on a behavioral level (5, 19, 24). These results draw on a wide area of research investigating the aberrant processing of pleasurable stimuli (i.e., “anhedonic symptoms”) in patients with psychiatric disorders, highlighting the ventral striatum (35–38) and medial pre-frontal cortex (39–41) as core structures involved. Extending this notion, previous studies have revealed that the aberrant coupling between frontal and striatal brain regions might be specifically involved in a reduced capacity to endure positive emotions (42), reduced reward receipt (43), and reduced positive affect (44) in a variety of psychiatric disorders. In sum, these first results corroborate the ideas put forward in the literature on positive affect and reward in the psychiatric domain, showing that the processing and production of humor might be related to the interaction and integration of frontal and subcortical brain regions crucially involved in motivated behavior. Similarly, however, as has been shown with research on antidepressant treatment (44), the engagement of frontal and subcortical structures in the experience of positive emotions can be systematically varied with targeted interventions. In the following, we sought to highlight the nature and efficacy of recent approaches to target both cognitive and emotional aspects of humor impairments in psychiatric disorders.

## Therapeutic Use of Humor in Psychiatry

In recent years, the interest in innovative therapy methods focusing on the patients resources and positive emotions has increased among psychiatrists and psychotherapists (45). Within the so-called “third wave” of behavioral therapy, numerous new transdiagnostic procedures (e.g., mindfulness-based cognitive therapy, schema therapy, etc.) have been developed, each with a strong focus on emotional processes. In this context, humor has been rediscovered as a therapeutic tool with interventions using various ways to make use of the therapeutic effects of humor. These accounts range from implementing the passive consumption of humorous material (e.g., via movies or clown doctors) in clinical day care, to the active use of humor via a systematic training of stand-up comedy skills or humor abilities (11, 13). Notably, however, the evaluation of these procedures is still in its infancy in many cases, especially with regard to the availability of randomized controlled studies and the consideration of findings from neurobiological research. In the following, we will (i) highlight how humor can be implemented as an element of therapy in the psychiatric domain and (ii) review the current state of research addressing the efficacy of humor interventions.

### Humor as an Element of Therapy

The use of humor has been described as an important aspect of psychotherapy across a wide range of orientations, including cognitive-behavioral (46) and family therapy (47). However, the techniques used to implement humor in the process of therapy vary widely (48, 49). One prominent way to include humor in daily psychiatric care is via the implementation of manual-based systematic group interventions addressing humor abilities (11–13). This approach is inspired by the social nature of humor and laughter, known to enhance socially rewarding relations. Since impairments in social functioning are a vital aspect in variety of psychiatric disorders, this approach seems to be the most promising for an efficient implementation in psychiatric care (49), while bearing the potential for a systematic evaluation of efficacy.

### Efficacy of Systematic Humor Interventions

In general, strength-based positive interventions have been shown to improve a variety of psychological factors in healthy subjects (50). Specifically, the effects of systematic humor interventions include the reduction of depressive and anxiety symptoms, improvement of self-esteem, enhanced humor production and appreciation, and improvements of social skills and social communication (12, 13). In a variety of individual investigations, humor interventions have been shown to strengthen the above variables in healthy subjects (51), geriatric patients (52, 53) and patients with mental disorders (11, 13), indicating a broad range of possible applications. However, the specific mechanisms on which their efficacy is based are currently unknown. Particularly, there is a fundamental lack of randomized controlled trials (i.e., using strong control conditions) in the psychiatric domain, testing for the specific influence of humor on variables of social and emotional functioning. Furthermore, although individual functional imaging studies on the neurobiological effects of social interventions in patients with schizophrenia indicate a normalization of fronto-cortical neural networks (54, 55), it is not yet known to what extent the altered humor processing in patients can be influenced with regard to its neural basis. Notably, however, a better understanding of pathophysiologically relevant processes could indeed provide essential insights into possible approaches for further therapeutic interventions, e.g., in terms of identifying target regions for neurostimulation procedures (56).

## Future Directions

Humor-based interventions have proven feasible in healthy individuals as well as clinical samples to contribute to psychological well-being and to the effectiveness of humor as a coping mechanism. These findings are in line with research suggesting that other positive psychological interventions are effective in increasing happiness and improving symptoms of mental disorders such as depression (57). While these findings may be encouraging for mental health practitioners looking to utilize patients' personal and social resources, additional high-quality peer-reviewed studies in diverse clinical populations are needed to strengthen the evidence-base for humor interventions. In particular, investigating the long-term effects of humor interventions and the degree of transfer of skills into everyday life is of great relevance, as the existing studies have mainly reported short-term positive outcomes, but the challenge is for these benefits to remain over a longer period of time. Future studies in clinical samples should also consider pre-intervention patient characteristics in more detail e.g., duration of illness, neurocognitive and psychosocial functioning, severity of symptoms and their impact on sense of humor measures, to determine whether patients with greater of fewer impairments will benefit the most or the least from humor interventions. Likewise, research into the length, intensity and focus (i.e., working on specific aspects vs. all aspects of the sense of humor) of humor interventions as well as the context in which they are provided (e.g., inpatient/outpatient settings, online interventions, individual vs. group interventions) is warranted, as differences in individual patient needs may affect their effectiveness.

In order to understand the mode of action of humor interventions in health and disease, brain imaging methods provide the opportunity to study processes associated with cognitive and emotional aspects of humor processing as well as intervention effects on these variables. To date no study has addressed the neural effects of humor interventions, although this is of particular relevance to put the advancement of the field of applied humor in therapy on a sound neurobiological basis. Future developments might include the evaluation of the neurobiological effects of humor vs. established interventions (e.g., mindfulness-based therapy, social skills trainings) as well as combinations of humor interventions with medication, brain stimulation methods or other (positive) psychological interventions. A better understanding of how humor and humor interventions affect mental health may thus inform the development and application of such resource-oriented approaches.

## Data Availability Statement

The original contributions presented in the study are included in the article/supplementary material, further inquiries can be directed to the corresponding author.

## Author Contributions

PB and IF wrote the first version of the manuscript. FB edited further versions of the manuscript. All authors contributed to the article and approved the submitted version.

## Conflict of Interest

The authors declare that the research was conducted in the absence of any commercial or financial relationships that could be construed as a potential conflict of interest.
